# Statin Decreases *Helicobacter pylori* Burden in Macrophages by Promoting Autophagy

**DOI:** 10.3389/fcimb.2016.00203

**Published:** 2017-01-17

**Authors:** Wei-Chih Liao, Mei-Zi Huang, Michelle Lily Wang, Chun-Jung Lin, Tzu-Li Lu, Horng-Ren Lo, Yi-Jiun Pan, Yu-Chen Sun, Min-Chuan Kao, Hui-Jing Lim, Chih-Ho Lai

**Affiliations:** ^1^Graduate Institute of Clinical Medical Science, China Medical UniversityTaichung, Taiwan; ^2^Department of Pulmonary and Critical Care Medicine, China Medical University HospitalTaichung, Taiwan; ^3^Department of Medical Laboratory Science and Biotechnology, China Medical UniversityTaichung, Taiwan; ^4^Department of Microbiology and Immunology, Graduate Institute of Biomedical Sciences, College of Medicine, Chang Gung UniversityTaoyuan, Taiwan; ^5^Graduate Institute of Basic Medical Science, School of Medicine, China Medical UniversityTaichung, Taiwan; ^6^Department of Urology, University of Texas Southwestern Medical CenterDallas, TX, USA; ^7^Department of Medical Laboratory Science and Biotechnology, Fooyin UniversityKaohsiung, Taiwan; ^8^Department of Laboratory Medicine, Chang Gung Memorial HospitalTaoyuan, Taiwan; ^9^Department of Nursing, Asia UniversityTaichung, Taiwan; ^10^Department of Pediatrics, Molecular Infectious Disease Research Center, Chang Gung Children's Hospital and Chang Gung Memorial HospitalTaoyuan, Taiwan

**Keywords:** autophagy, cholesterol, *Helicobacter pylori*, HMG-CoA reductase, statin

## Abstract

Statins, 3-hydroxy-3-methyl-glutaryl-coenzyme A (HMG-CoA) reductase inhibitors, have been found to provide protective effects against several bacterial infectious diseases. Although the use of statins has been shown to enhance antimicrobial treated *Helicobacter pylori* eradication and reduce *H. pylori*-mediated inflammation, the mechanisms underlying these effects remain unclear. In this study, *in vitro* and *ex vivo* macrophage models were established to investigate the molecular pathways involved in statin-mediated inhibition of *H*. *pylori*-induced inflammation. Our study showed that statin treatment resulted in a dose-dependent decrease in intracellular *H*. *pylori* burden in both RAW264.7 macrophage cells and murine peritoneal exudate macrophages (PEMs). Furthermore, statin yielded enhanced early endosome maturation and subsequent activation of the autophagy pathway, which promotes lysosomal fusion resulting in degradation of sequestered bacteria, and in turn attenuates interleukin (IL)-1β production. These results indicate that statin not only reduces cellular cholesterol but also decreases the *H*. *pylori* burden in macrophages by promoting autophagy, consequently alleviating *H*. *pylori*-induced inflammation.

## Introduction

*Helicobacter pylori* is a Gram-negative microaerophilic spirochete that colonizes the human stomach and is estimated to have infected greater than half of the global population (Marshall, 2002). Persistent *H*. *pylori* infection is associated with several upper gastrointestinal disorders such as gastritis, peptic ulcers, and gastric adenocarcinoma (Wroblewski et al., 2010).

Although *H. pylori* is generally considered an intracellular pathogen, this organism lives in the mucosal layer and tightly adheres to the gastric epithelial surface. Notably, virulent strains of *H. pylori*, can delay uptake and promote the formation of megasomes within macrophages, which comprises a crucial feature of *H. pylori*-induced pathogenesis (Allen et al., 2000). Moreover, cholesterol-α-glucosyltransferase, which is responsible for cholesterol glucosylation in macrophages, was found to contribute to the protection of *H. pylori* from phagocytosis (Wunder et al., 2006). These lines of evidence suggest that *H. pylori* can survive intracellularly within specific compartments of macrophages to avoid phagocytosis-mediated killing.

The inhibitors of 3-hydroxy-3-methyl-glutaryl-coenzyme A (HMG-CoA) reductase, commonly known as statins, are widely prescribed for lowering serum cholesterol (Armitage, 2007). Notably, statins have also been shown to reduce the risk of severe bacterial infections, including infections by *Chlamydia pneumoniae* (Erkkilä et al., 2005), *Clostridium difficile* (Motzkus-Feagans et al., 2012), *Staphylococcus aureus* (Chow et al., 2010), and *Streptococcus pneumoniae* (Boyd et al., 2012). However, the immunomodulatory properties of statins provide only a partial explanation for the mechanism by which these compounds inhibit bacterial infections (Jain and Ridker, 2005).

The human immune system employs various mechanisms to inhibit bacterial infections. While autophagy is a cell process that typically functions as a recycling pathway, degrading nonfunctional and unnecessary components and rearranging these components to support cellular survival (Mariño et al., 2014), this process was also found to contribute to immune defense by degrading invading pathogens (Mizushima et al., 2008; Zhao et al., 2008). As such, these findings indicate that stimulation of cellular autophagy may attenuate *H*. *pylori*-induced pathogenesis (Yang and Chien, 2009).

Antimicrobial agents, particularly a triple therapy regimen consisting of a proton-pump inhibitor, amoxicillin, and clarithromycin, are the most effective means of eradicating *H*. *pylori* infections (O'Connor et al., 2013). Although the cure rate varies between countries, the triple therapy regimen remains the recommended treatment for *H*. *pylori* infection (O'Connor et al., 2013). Notably, the administration of this triple therapy regimen along with statins has been shown to accelerate the clearance of *H*. *pylori* and reduce *H*. *pylori*-related inflammation (Tariq et al., 2007; Yamato et al., 2007; Nseir et al., 2012). However, the molecular mechanisms underlying the regulatory effects of statins on *H*. *pylori*-induced pathogenesis require further investigation. In this study, we first hypothesized that statins may influence the immune response via upregulation of autophagy and attenuation of *H*. *pylori*-induced inflammation. We utilized *in vitro* and *ex vivo* macrophage models of *H*. *pylori* infection to investigate the mechanism underlying the statin-mediated mitigation of *H*. *pylori* pathogenesis. We also explored how statin influences the bacterial burden and reduces inflammation by upregulating cellular autophagy and consequently alleviating *H*. *pylori*-associated pathogenesis.

## Materials and methods

### Antibodies and reagents

The light chain 3 (LC3)-specific monoclonal antibody was purchased from Cell Signaling Technology (Danvers, MA), while the rabbit antibodies against SQSTM1/p62 and beclin-1 were purchased from GeneTex (Irvine, CA). The rabbit anti-early endosome antigen-1 (EEA-1), anti-lysosome-associated membrane protein-1 (LAMP-1), and mouse monoclonal anti-β-actin antibodies were purchased from Abcam (Cambridge, UK), Abgent (San Diego, CA), and Santa Cruz Biotechnology (Dallas, TX), respectively. Simvastatin was purchased from Sigma-Aldrich (St. Louis, MO).

### Bacterial and cell culture

*H*. *pylori* 26695 (ATCC 700392) was cultured on 10% sheep blood agar plates in a microaerophilic environment (10% CO_2_, 5% O_2_, and 85% N_2_) at 37°C. Cultures were incubated for 24–36 h to achieve optimum microbial activity (Lai et al., 2005). Murine RAW264.7 macrophages (ATCC TIB-71) were cultured in Dulbecco's Modified Eagle Medium (DMEM) supplemented with 10% endotoxin-free fetal bovine serum (HyClone, Logan, UT).

### Analysis of cellular cholesterol and cytotoxicity

RAW264.7 cells were treated with simvastatin (0, 5, or 10 μM) at 37°C for 24 h. Untreated cells were utilized as a control. The cellular cholesterol content of each treatment group was then evaluated using an Amplex Red cholesterol assay kit (Molecular Probes, Carlsbad, CA). The percentage of cellular cholesterol was determined as follows: (fluorescence of treated cells obtained from a standard curve/total fluorescence of untreated cells) × 100%. Meanwhile, the viability of RAW264.7 cells was determined via the trypan blue exclusion assay, as described previously (Lai et al., 2008).

### Preparation of murine peritoneal exudate macrophages (PEMs)

PEMs isolated from C57BL/6 mice were used to investigate the effects of simvastatin on *H*. *pylori*-induced autophagy. Mice were maintained at the Animal Center of Chang Gung University (Taoyuan, Taiwan). All procedures were performed according to the “Guide for the Care and Use of Laboratory Animals” (NRC, USA) and were approved by the Animal Experimental Committee of Chang Gung University. PEMs were prepared after euthanasia by lavaging mice with an intraperitoneal injection of 3% thioglycolate, as described previously (Lu et al., 2012a). Harvested cells were then seeded into 24-well tissue culture plates and incubated at 37°C for 2 h, after which non-adherent cells were removed. Adherent cells were then treated with simvastatin and/or *H*. *pylori* and subjected to bacterial intracellular survival assay and western blot analyses.

### Phagocytosis assay

RAW264.7 cells were treated with PBS or simvastatin (0, 5, or 10 μM) for 8 h and incubated with latex beads (IgG-FITC complex) at a ratio of 1:100, according to the manufacturer's instructions (Cayman, Ann Arbor, MI). After incubation for 1 h, the treated cells were washed with PBS, fixed in 3.7% paraformaldehyde, and then subjected to flow cytometry analysis.

### Bacterial adhesion assay

The numbers of cell-associated bacteria were measured as described previously (Lai et al., 2008). Briefly, RAW264.7 cells were treated with PBS or 10 μM simvastatin for 8 h and then infected with *H. pylori* at a multiplicity of infection (MOI) of 100 for 6 h. Infected cells were washed three times to remove unbound bacteria and then lysed with distilled water for 10 min. Lysates were diluted in PBS, then plated onto Brucella blood agar plates and cultured for 3–5 days. Viable bacteria were counted and expressed as colony-forming units (CFU).

### Bacterial intracellular survival assay

RAW264.7 cells and murine PEMs and cells were not treated or treated with 10 μM simvastatin for 8 h and then infected with *H*. *pylori* (MOI = 100) for 16 h. Cells were treated with gentamycin (100 μg/ml) to eradicate the extracellular bacteria (Lai et al., 2006). The *H. pylori*-infected cells were washed with PBS three times and then incubated in sterile water at 37°C for 10 min to osmotically disrupt the cell membrane. The resulting lysates were then diluted in PBS and applied to Brucella blood agar plates. Viable *H*. *pylori* colonies were enumerated after 3–5 days of incubation and were presented as CFU.

### Western blot analysis

To analyze protein expression levels, RAW264.7 cells or PEMs were treated with simvastatin (0, 5, or 10 μM) at 37°C for 8 h prior to infection with *H. pylori* for 16 h. Cell lysates were prepared and boiled with sample dye at 100°C for 5 min. Samples were separated by 10–12% sodium dodecyl sulfate-polyacrylamide gel electrophoresis (SDS-PAGE) and transferred to polyvinylidene fluoride membranes (Millipore, Billerica, MA). Membranes were blocked by incubation in TBS-T (Tris-buffered saline containing 0.1% Tween 20) containing 5% skim milk at room temperature for 1.5 h, and then probed with primary antibodies specific to autophagy-related proteins or β-actin (0.2 μg/ml), respectively, at 4°C overnight. Probed membranes were washed with TBS-T and incubated with horseradish peroxidase-conjugated secondary antibodies (0.1 μg/ml) (Santa Cruz Biotechnology) at room temperature for 1 h, and proteins of interests were visualized using ECL™ western blotting Detection Reagents (GE Healthcare, Little Chalfont, UK), and an ImageQuant LAS-4000 system (GE Healthcare). Protein expression levels were quantified using UN-SCAN-IT gel 6.1 software (Silk Scientific Corporation, Orem, UT).

### Immunofluorescence microscopy

To visualize the co-localization of autophagosome fused with lysosome, cells were subjected to immunofluorescence microscopy analysis (Parihar et al., 2014). RAW264.7 cells (2 × 10^6^) were seeded on cover glass in 6-well plates, treated with 10 μM simvastatin, and co-incubated with *H*. *pylori* (MOI = 100) for 16 h. To observe the early to late stages of autophagosome formation, the cells were probed using a Cyto-ID™ autophagy detection kit (Enzo Life Sciences, Villeurbanne, France; Chan et al., 2012). Meanwhile, the formation of lysosome was observed using a Cell Navigator™ Lysosome Staining Kit (AAT Bioquest, Sunnyvale, CA). Fluorescence signals from *H*. *pylori*-infected macrophages were visualized by confocal laser-scanning microscopy (Zeiss LSM 780; Carl Zeiss, Oberkochen, Germany).

### Enzyme-linked immunosorbent assay (ELISA)

After treating with 10 μM simvastatin and infecting with *H*. *pylori*, RAW264.7 cell supernatants were collected and interleukin (IL)-1β secretion was analyzed using a mouse IL-1β ELISA kit, according to the manufacturer's instructions (Invitrogen, Waltham, MA).

### Statistical analysis

Experimental results are expressed as means ± standard errors of the mean (SEM). Differences in results between groups were evaluated using Student's *t*-tests. For analyses of variance, one-way analysis of variance (ANOVA) was utilized. *P* < 0.01 were considered statistically significant. Statistical analyses were performed using SPSS version 11.0 software (SPSS Statistics, Inc., Chicago, IL).

## Results

### Statin reduces the intracellular burden of *H. pylori* in macrophages

**Figure 1:**
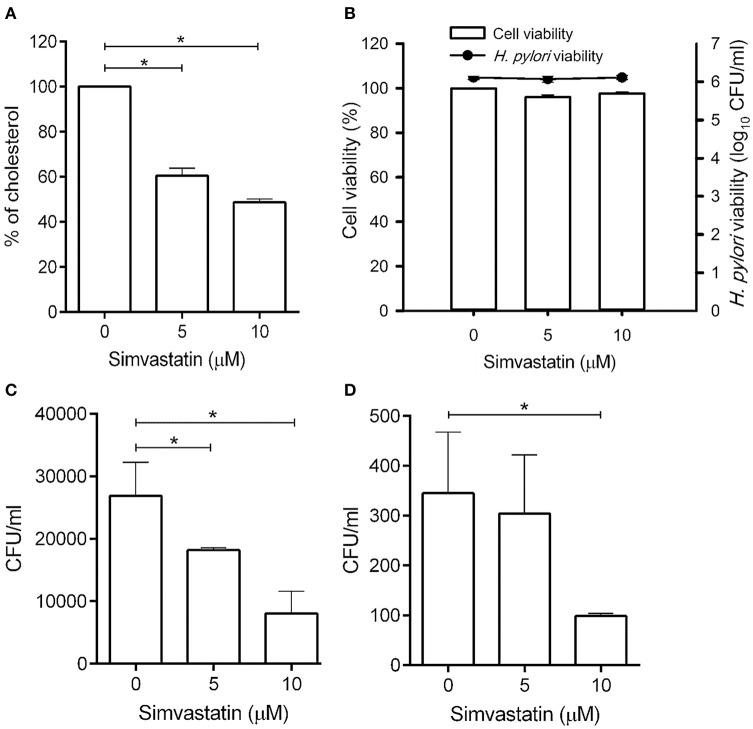
**Statin treatment reduces cellular cholesterol and decreases the intracellular bacterial burden in macrophages. (A)** RAW264.7 cells were treated with various concentrations of simvastatin (0, 5, or 10 μM) for 24 h and subjected to cellular cholesterol assay analysis. **(B)** RAW264.7 and *Helicobacter pylori* cultures were treated with 0, 5, or 10 μM simvastatin for 24 h. Macrophage cell viability was then assessed by trypan blue exclusion assay analysis and bacterial viability was determined by counting the number of colony-forming units (CFU) for each treatment group on blood agar plates. **(C)** RAW264.7 cells and **(D)** murine peritoneal exudate macrophages (PEMs) were pretreated with simvastatin (0, 5, or 10 μM) for 8 h and infected with *H*. *pylori* at a MOI of 100 for 16 h. The numbers of viable intracellular bacteria were then determined via gentamicin protection assays and were expressed as the number of viable CFU. Statistical significance was evaluated using Student's *t*-test. ^*^P < 0.01.

Statins are known as inhibitors of HMG-CoA reductase, which is the rate-limiting enzyme of the mevalonate pathway (Armitage, 2007). In this study, we first evaluated whether simvastatin is capable of reducing the cellular cholesterol of macrophages. As shown in Figure 1A, there was a dose-dependent reduction in the level of cellular cholesterol in RAW264.7 macrophage cells treated with simvastatin (Figure 1A). We then assessed whether simvastatin treatment affected macrophage or bacterial viability. Notably, both macrophages and *H*. *pylori* remained viable, even after treatment with 10 μM simvastatin (Figure 1B). These results indicate that simvastatin reduces the levels of cellular cholesterol in macrophages without affecting cell viability or bacterial survival.

To further validate whether statin-treated macrophages function similarly to untreated macrophages, RAW264.7 cells were untreated or treated with statin (10 μM) and subjecting to the latex bead phagocytosis assay. As shown in Figure S1, macrophages treated with statin exhibited similar levels of phagocytosis to the untreated cells. Likewise, statin treatment had no significant effect on extracellular bacterial adhesion (Figure S2). Together, these results demonstrate that macrophages retain their normal functions in the presence of statins.

To investigate whether simvastatin inhibits *H*. *pylori* survival in macrophages, RAW264.7 cells were pretreated with simvastatin for 8 h and infected with *H*. *pylori* (MOI = 100) for an additional 16 h. As shown in Figure 1C, treatment with 5 and 10 μM simvastatin resulted in significant dose-dependent reductions in *H*. *pylori* burden in RAW264.7 cells. To further explore the inhibitory effects of simvastatin on intracellular bacterial survival, we performed an *ex vivo* analysis using murine PEMs isolated from C57BL/6 mice. As observed in RAW264.7 cells, PEMs that were pretreated with 5 or 10 μM simvastatin for 8 h exhibited significant reductions in *H*. *pylori* burden after 16 h of infection compared to the control population (Figure 1D). These results demonstrate that simvastatin decreases the intracellular *H*. *pylori* burden in both in RAW264.7 and primary murine PEMs in a dose-dependent manner.

### Statin promotes *H. pylori*-induced autophagy in macrophages

**Figure 2:**
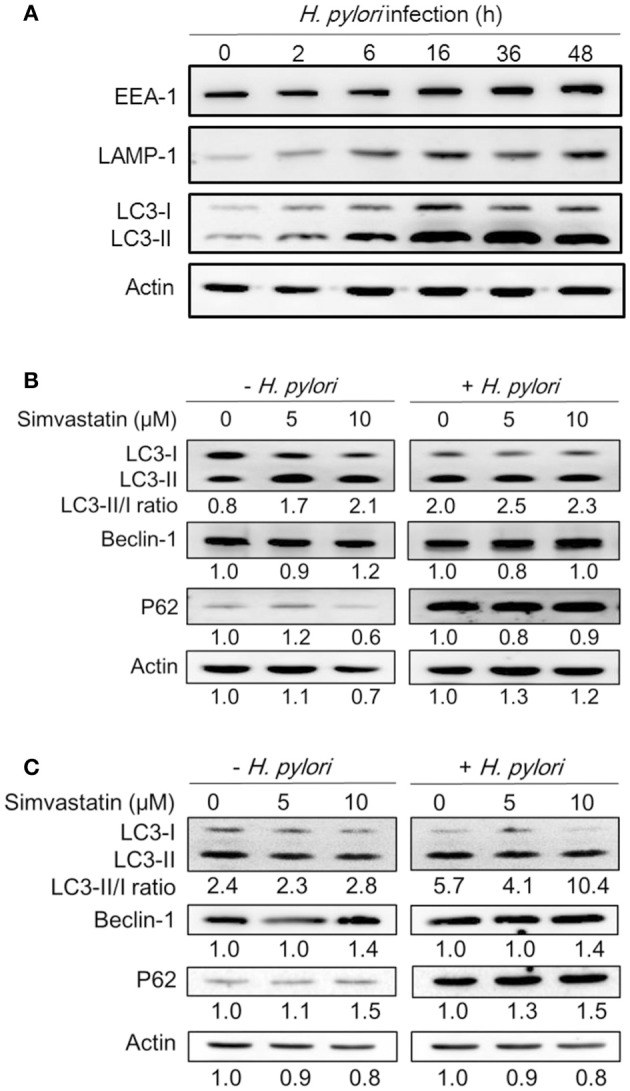
**Statin increases ***Helicobacter pylori***-induced autophagy in macrophages. (A)** RAW264.7 cells were infected with *H. pylori* at a MOI of 100 for the indicated times (0, 2, 6 16, 36, and 48 h). The expression levels of proteins were then evaluated by western blot analysis. **(B)** RAW264.7 cells and **(C)** peritoneal exudate macrophages (PEMs) were pretreated with simvastatin (0, 5, or 10 μM) for 8 h and then infected with *H. pylori* at a MOI of 100 for an additional 16 h. Cell lysates were then prepared and subjected to western blot analysis for detection of autophagy-associated proteins. β-actin expression levels were used as the protein loading control. Representative western blot results from one of two independent experiments are shown. The expression level of each protein was quantified by analysis of signal intensity, and normalized with that of β-actin. The expression level of each respective protein, relative to that of the control group, is indicated at the bottom of each lane.

Because treatment of cells with cholesterol-lowering agents has been shown to induce autophagy (Cheng et al., 2006; de Chastellier and Thilo, 2006), we investigated whether statin influences the immune response by upregulating autophagy and attenuating *H. pylori*-induced inflammation. To address this question, we established a macrophage infection model to explore the mechanisms involved in the inhibition of *H. pylori*-induced inflammation by statin. Following the induction of autophagy, the microtubule-associated protein LC3 is converted from LC3-I to LC3-II. As such, the expression of LC3-II is considered a marker of autophagy (Klionsky et al., 2012). We therefore evaluated the expression levels of this protein, as well as those of EEA-1, an early marker localized on phagosomal membranes, and LAMP-1, a marker localized on lysosomal membranes (Fratti et al., 2001; Huynh et al., 2007; Parihar et al., 2014), in RAW264.7 cells infected with *H. pylori* for 0–48 h by western blot analysis. As shown in Figure 2A, there was a gradual increase in the levels of *H. pylori*-induced EEA-1 and LAMP-1 expression, and a concurrent increase in the conversion of LC3-I to LC3-II, over the course of the infection. Peak levels of conversion from LC3-I to LC3-II were observed between 16 and 36 h. Next, we evaluated the expression of the autophagy-related proteins beclin-1 and SQSTM1/p62, which are known to participate in the initiation of autophagy with LC3-II (Kang et al., 2011; Levine et al., 2011). RAW264.7 cells treated with simvastatin (0, 5, or 10 μM) and infected with *H. pylori* for 16 h exhibited slightly increased expression of beclin-1 and p62 (Figure 2B). To confirm these findings, we subsequently analyzed the effects of simvastatin on PEMs isolated from C57BL/6 mice. As shown in Figure 2C, we observed increased levels in the conversion of LC3-I to LC3-II, as well as increased expression of beclin-1 and p62, in *H. pylori*-infected PEMs treated with simvastatin, compared to untreated cells. These results suggest that simvastatin treatment not only reduces cellular cholesterol but also promotes *H. pylori*-induced autophagy in macrophages.

### Statin facilitates autophagosome formation in *H. pylori*-infected macrophages

**Figure 3:**
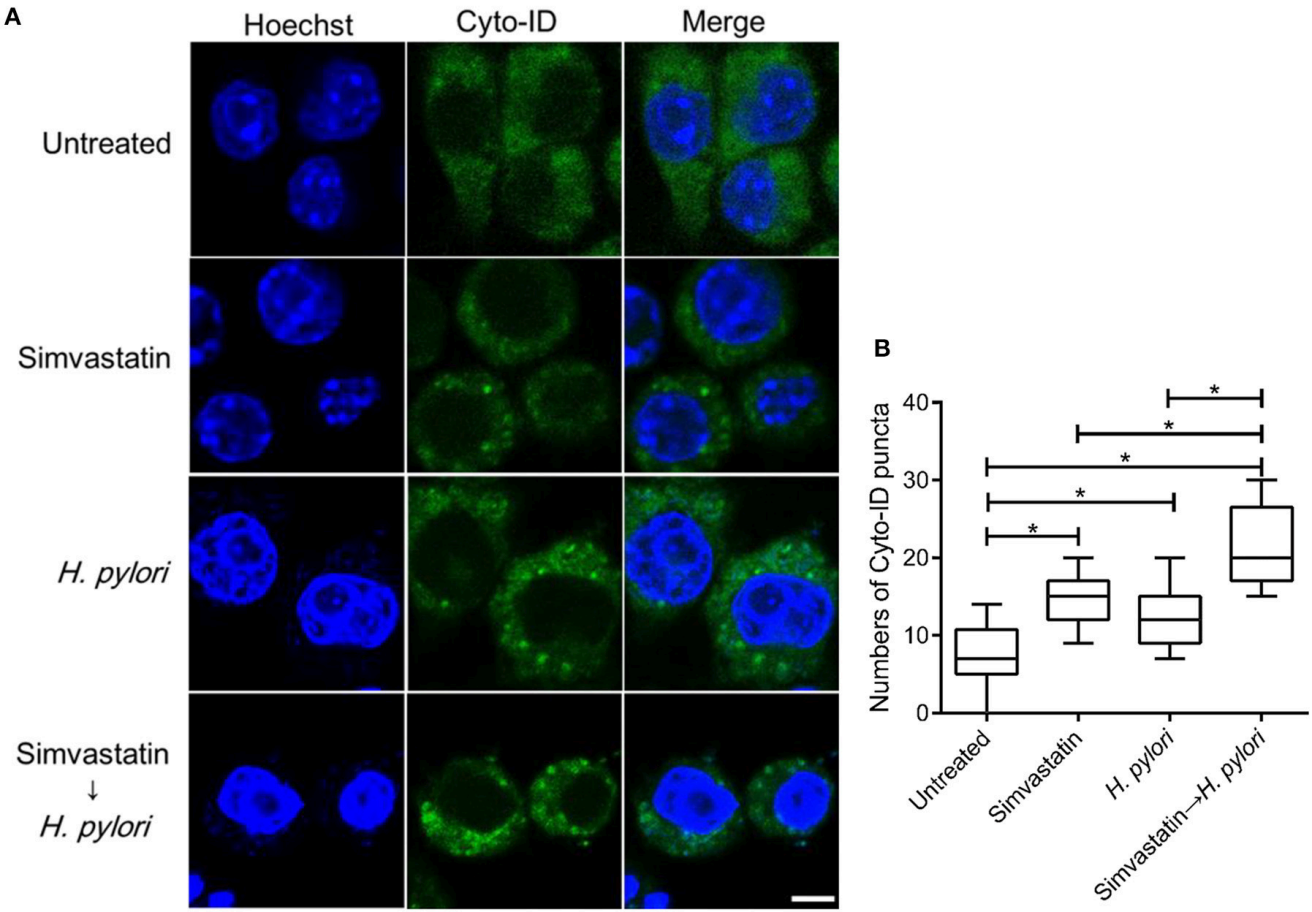
**Statin enhances autophagosome formation in ***Helicobacter pylori***-infected macrophages**. **(A)** Untreated RAW264.7 cells and cells treated with 10 μM simvastatin were infected with or without *H. pylori* at a MOI of 100. After incubation for 16 h, the cells were fixed and stained with Cyto-ID and Hoechst 33342 for detection of autophagosomes (green) and visualization of nuclei (blue), respectively. Stained cells were analyzed by confocal microscopy. Scale bar, 5 μm. **(B)** Box plots summarizing the number of Cyto-ID puncta in each cell (50 cells were evaluated per sample). Statistical significance was evaluated using Student's *t*-test. ^*^P < 0.01.

To further evaluate the effects of simvastatin on macrophages, RAW264.7 cells were stained with Cyto-ID autophagy green dye for visualization of autophagosomes and examined by confocal microscopy. The control group (untreated and uninfected) exhibited faint Cyto-ID green fluorescence (puncta-formation; Figure 3A), while cells treated with simvastatin or *H. pylori* alone showed only moderate staining. In contrast, cells that were treated with simvastatin and subsequently infected with *H. pylori* exhibited significantly increased autophagosome formation compared to both the control group and to the cells treated with simvastatin or *H. pylori* alone (Figure 3B). These results demonstrate that simvastatin enhances *H. pylori*-induced autophagosome formation in macrophages.

### Statin promotes autophagosome and lysosome fusion in *H. pylori*-infected macrophages

**Figure 4:**
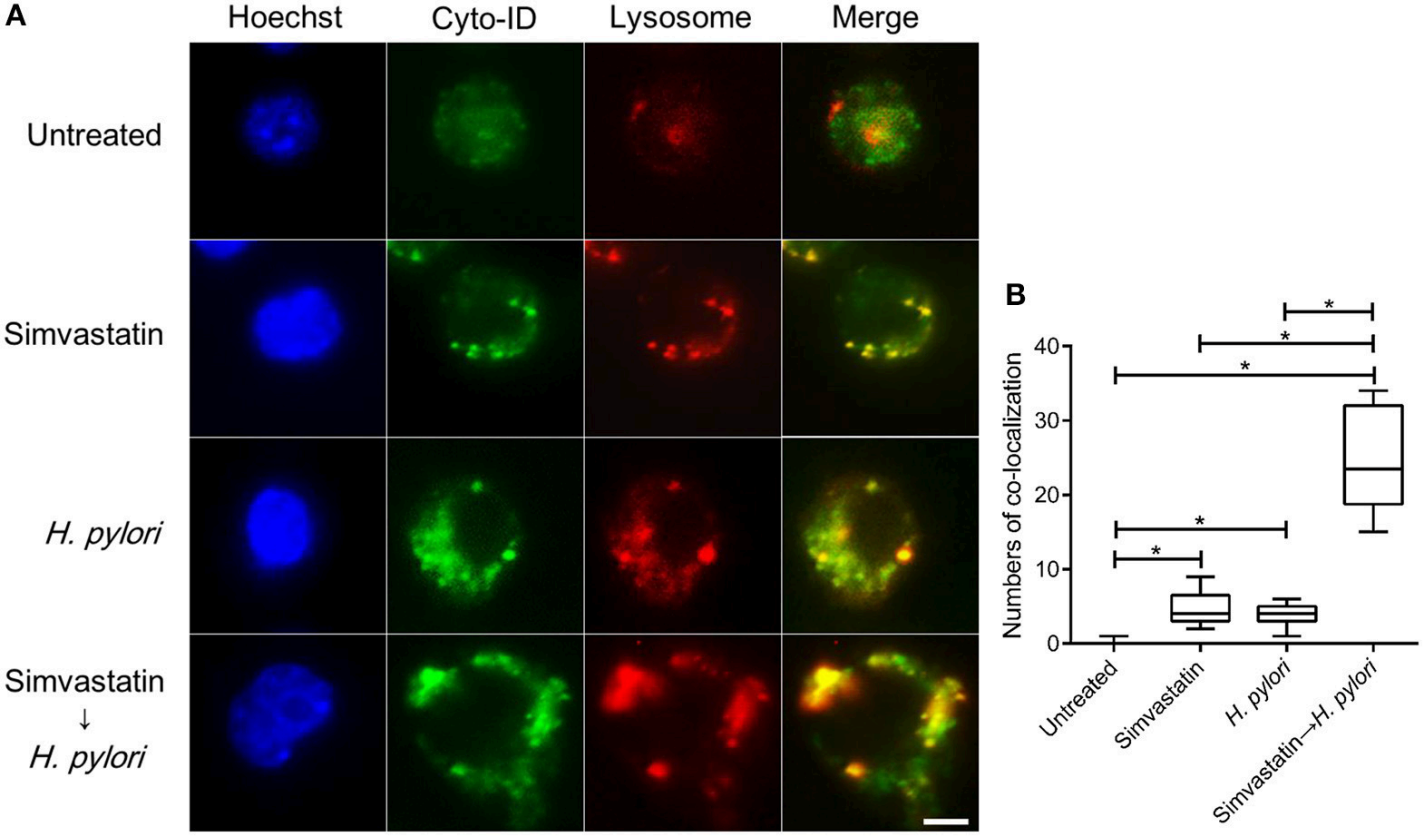
**Statin facilitates the fusion of autophagosomes and lysosomes in ***Helicobacter pylori***-infected macrophages. (A)** Untreated RAW264.7 cells and cells treated with 10 μM simvastatin were infected with or without *H. pylori* at a MOI of 100 for 16 h. Cells were then fixed, stained with Cyto-ID (green), Lysosome Staining (red), and Hoechst 33342 (blue) for visualization of autophagosomes, lysosomes, and nuclei, respectively, and analyzed by confocal microscopy. Scale bar, 5 μm. **(B)** Box plots summarizing the numbers of co-localized Cyto-ID puncta and lysosomes in each cell (50 cells were evaluated per sample). Statistical significance was evaluated using Student's *t*-test. ^*^P < 0.01.

**Figure 5:**
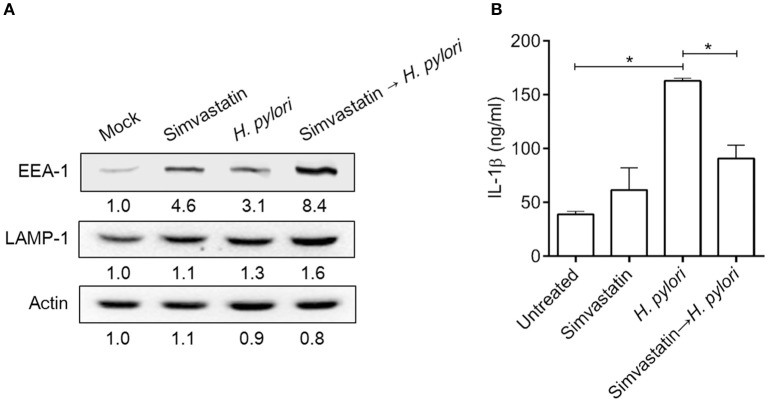
**Statin induces lysosome formation and mitigates proinflammatory cytokine production in ***Helicobacter pylori***-infected macrophages**. RAW264.7 cells were treated with 10 μM simvastatin for 8 h and then infected with *H. pylori* at a MOI of 100 for an additional 16 h. **(A)** The expression levels of EEA-1 and LAMP-1 were analyzed by western blot. Representative western blot results from one of two independent experiments are shown. The expression level of each protein was quantified by analysis of signal intensity, and normalized to that of β-actin. The expression level of each respective protein, relative to that of the control group, is indicated at the bottom of each lane. **(B)** The levels of IL-1β present in each culture supernatant were determined by enzyme-linked immunosorbent assay (ELISA). Statistical significance was evaluated using Student's *t*-test. ^*^P < 0.01.

Mature autophagosomes fuse with lysosomes to form autolysosomes, which contain lysosomal enzymes capable of degrading sequestered bacteria (Deen et al., 2013). We thus investigated whether statin treatment promotes bacterial degradation in macrophages by inducing autophagosome maturation and fusion with lysosomes. As shown in Figure 4A, RAW264.7 cells treated with either simvastatin or *H. pylori* alone exhibited little co-localization of Cyto-ID and lysosomal fluorescence within the cytoplasm. Conversely, cells treated with simvastatin and then infected with *H. pylori* exhibited marked co-localization of Cyto-ID and lysosomes (Figure 4B). We also tested whether statin treatment promoted *H. pylori*-induced lysosome formation. As shown in Figure 5A, treatment with both simvastatin and *H. pylori* resulted in increases in EEA-1 and LAMP-1 expression, compared with cells exposed to simvastatin or *H. pylori* alone. We then sought to analyze whether statin reduces the bacterial burden, and whether this had any effect on IL-1β production. The induction of IL-1β was dramatically increased in cells infected with *H. pylori* alone (Figure 5B). In contrast, when cells were treated with 10 μM simvastatin and then infected with *H. pylori*, there was a significant decrease in the IL-1β secretion compared with the cells exposed to *H. pylori*. Collectively, our results reveal that statin treatment facilitated *H. pylori*-induced autophagy, and subsequently promoted autophagosome maturation and fusion with lysosomes, thereby reducing the bacterial burden in macrophages and ameliorating the inflammatory response.

## Discussion

Cholesterol-enriched microdomains, which provide platforms for microbial infection, are thought to be associated with various infectious bacterial and viral diseases (Lin et al., 2015). In particular, cholesterol plays a crucial role in *H. pylori* cell invasion and virulence (Lai et al., 2013). Depletion of cellular cholesterol not only attenuates *H. pylori*-induced pathogenesis (Hutton et al., 2010; Lai et al., 2011) but also promotes autophagy (Cheng et al., 2006), which has been shown to contribute to immune defense against invading pathogens (Levine et al., 2011; Deretic et al., 2013; Lai et al., 2015). Statins, inhibitors of HMG-CoA reductase that are widely prescribed for lowering serum cholesterol, have also been employed to reduce the risk of certain bacterial infections (Chow et al., 2010; Nseir et al., 2010; Boyd et al., 2012; Motzkus-Feagans et al., 2012). The *in vitro* and *ex vivo* approaches utilized in this study provided evidence that statin treatment results in reduced bacterial burden in macrophages, and consequently attenuation of *H. pylori* pathogenesis, via activation of autophagy. These findings suggest that such cholesterol modulation could comprise a novel therapeutic approach for controlling *H. pylori*-associated diseases.

Previous studies have shown that *H. pylori* actively delays its uptake by macrophages, inhibits phagosome maturation via a VacA and urease-dependent mechanism, and resides in large vacuoles called megasomes (Allen et al., 2000; Schwartz and Allen, 2006). Another unusual feature of *H. pylori* is a requirement for cholesterol, which is acquired from host cell membranes and incorporated into the bacterium as cholesteryl glucosides (Wunder et al., 2006). In addition, it has been reported that *H. pylori* encodes several virulence factors that exploit cholesterol to gain a foothold in the host niche (Murata-Kamiya et al., 2010; Lai et al., 2013). Meanwhile, surface molecules located within the cholesterol-rich microdomains of host cells sense and respond to *H. pylori* in an orchestrated manner (Lu et al., 2012b; Lin et al., 2016). As such, both host- and pathogen-derived factors play critical roles in disease progression. In previous studies, we demonstrated that reduced cellular cholesterol resulted in reduced VacA activity, as well as attenuated CagA-induced inflammation and decreased bacterial survival, in *H. pylori*-infected gastric epithelial cells (Lai et al., 2008, 2011; Wang et al., 2012). Specifically, reductions in cholesterol disrupt the integrity of lipid rafts, resulting in decreased type IV secretion system-mediated translocation of CagA into host cells (Hutton et al., 2010), thereby reducing downstream signaling and attenuating the inflammatory response (Lai et al., 2011). In the current study, we demonstrate that statin treatment yielded enhanced autophagy and reduced bacterial burdens in macrophages, followed by decreased levels of *H. pylori*-induced IL-1β production, suggesting that statin may attenuate *H. pylori*-induced pathogenesis via multiple mechanisms.

Autophagy is a process involving the degradation and recycling of intracellular components to provide cellular energy and maintain nutritional support for cell survival (Singh and Cuervo, 2011). Previous studies reported that *H. pylori* infection induces autophagosome formation, and that autophagic vesicles provide special niches for *H. pylori* multiplication in both gastric epithelial cells (Terebiznik et al., 2009) and macrophages (Wang et al., 2009). Although *H. pylori* was protected in autophagosomes during the early stages of infection, the bacteria were degraded within the resulting autolysosomes of both cell types after 24 h of infection (Chu et al., 2010; Wang et al., 2010). Consistent with these findings, peripheral blood monocytes (PBMCs) harboring a single nucleotide polymorphism within the coding sequence for Autophagy Related 16-Like 1 (ATG16L1; *ATG16L1300A*), which is essential for autophagosome formation, exhibited impaired autophagy and increased susceptibility to *H. pylori* infection (Raju et al., 2012). In contrast, PBMCs harboring the *ATG16L1300T* allele showed a superior autophagic response and exhibited increased *H. pylori* clearance compared with PBMCs carrying the *300A* allele. These data imply that induction of autophagy might comprise a general mechanism for restricting bacterial replication in phagocytes (Deen et al., 2013).

**Figure 6:**
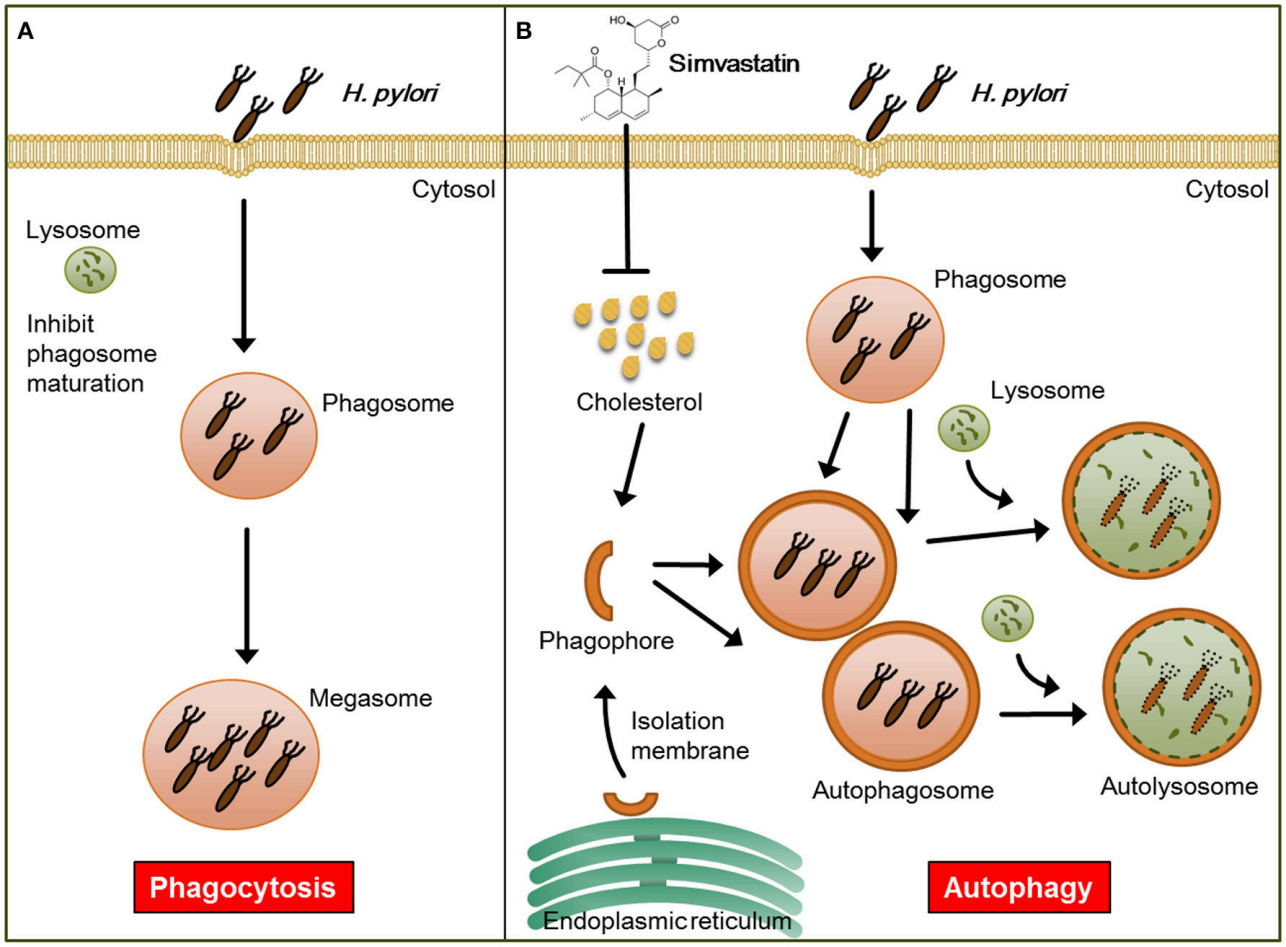
**Model for the mechanism by which statin enhances autophagy and alleviates pathogenic infection by ***Helicobacter pylori***. (A)** Infected macrophages harbor *H. pylori*, which inhibit phagosome maturation, within large vacuoles called megasomes. **(B)** Initiation of the autophagy pathway includes the translocation of the ULK complex from the cytosol to the endoplasmic reticulum (ER) and recruitment of the PI3K complex, which triggers the initiation of phagophore nucleation (Greenfield and Jones, 2013). This initiation process forms the isolation membranes, which in turn sequester cellular components and fuse to generate autophagosomes. Treatment of cells with simvastatin promotes the fusion of autophagosomes with lysosomes, leading to reduction in bacterial burdens and attenuation of *H. pylori*-induced inflammation.

In addition to lowering cellular cholesterol, statins were found to play a protective role in several bacterial infectious diseases. For example, while previous studies have established that *Listeria monocytogenes* triggers autophagy, resulting in reduced bacterial growth (Birmingham et al., 2007; Py et al., 2007), in a listeriolysin O (LLO)-dependent manner (Meyer-Morse et al., 2010), statin treatment was found to enhance the host defense against *L. monocytogenes* by targeting LLO-mediated phagosomal escape (Parihar et al., 2013). Furthermore, it was recently reported that statin therapy protects against *Mycobacterium tuberculosis* infection by enhancing autophagy and promoting phagosome maturation (Parihar et al., 2014). Consistent with these previous findings, our results demonstrate that statin treatment promoted autophagosome maturation and fusion with lysosomes, resulting in reduced bacterial burdens within macrophages, and thereby mitigated pathogenic infection by *H. pylori* (Figure 6).

There are several limitations to this study. First, only RAW264.7 macrophages and PEMs were utilized in our experiments; we have yet to test our findings in animal or human subjects. Likewise, the statin dosing regimens used in our cell-based study have yet to be evaluated *in vivo*. Therefore, further clinical investigations are required to clarify the link between statin use and regulation of autophagy. Such studies might pave the way for developing new strategies to control *H. pylori* infection.

In conclusion, the results of this investigation reveal that statin could potentially be used to decrease the intracellular burden of *H. pylori* in macrophages. In addition, statin enhanced early endosome maturation and subsequent activation of autophagy in macrophages, which promoted lysosomal fusion resulting in the degradation of sequestered *H. pylori*, followed by reductions in proinflammatory cytokine production. Future *in vivo* investigations and treatment regimens are needed to study the mechanism underlying the statin-mediated mitigation of *H. pylori* infection.

## Author contributions

Conception or design of this work: WL, MH, and C-HL. Experimental study: WL, MH, MW, C-JL, TL, H-RL, and H-JL. Data analysis and interpretation: YS, MK, and YP. Writing the manuscript: WL, MH, and C-HL. Final approval: all authors

## Funding

This work was supported by the Ministry of Science and Technology (104-2320-B-182-040 and 105-2313-B-182-001), Chang Gung Memorial Hospital (CMRPD1F0011-3, CMRPD1F0431-3, and BMRPE90), and the Tomorrow Medical Foundation.

### Conflict of interest statement

The authors declare that the research was conducted in the absence of any commercial or financial relationships that could be construed as a potential conflict of interest.

## Supplementary material

Figure S1 **Statin does not affect the phagocytotic activity of macrophages**. RAW264.7 cells treated with simvastatin (0 or 10 μM) for 8 h were incubated with latex beads, and phagocytosis activity was evaluated via flow cytometry analysis. The number at the right of each histogram represents the mean fluorescence intensity (MFI).

Figure S2 **Statin has no effect on extracellular bacterial adhesion to macrophages**. RAW264.7 cells untreated or treated with 10 μM simvastatin for 8 h were infected with *Helicobacter pylori* (MOI = 100) for 6 h, washed three times to remove unbound bacteria, and lysed by incubation in distilled water for 10 min. Cell lysates were then plated onto Brucella blood agar plates and cultured for 3–5 days. The results are expressed as the mean (± standard deviation) number of viable colony-forming units (CFU) obtained from three independent experiments.
