# Supplementary figures and images for: Statin Decreases Helicobacter pylori Burden in Macrophages by Promoting Autophagy
Source: Front Cell Infect Microbiol. 2017 Jan 17;6:203. doi: 10.3389/fcimb.2016.00203 (PMC5239775; doi:10.3389/fcimb.2016.00203)

**Fig. S1**

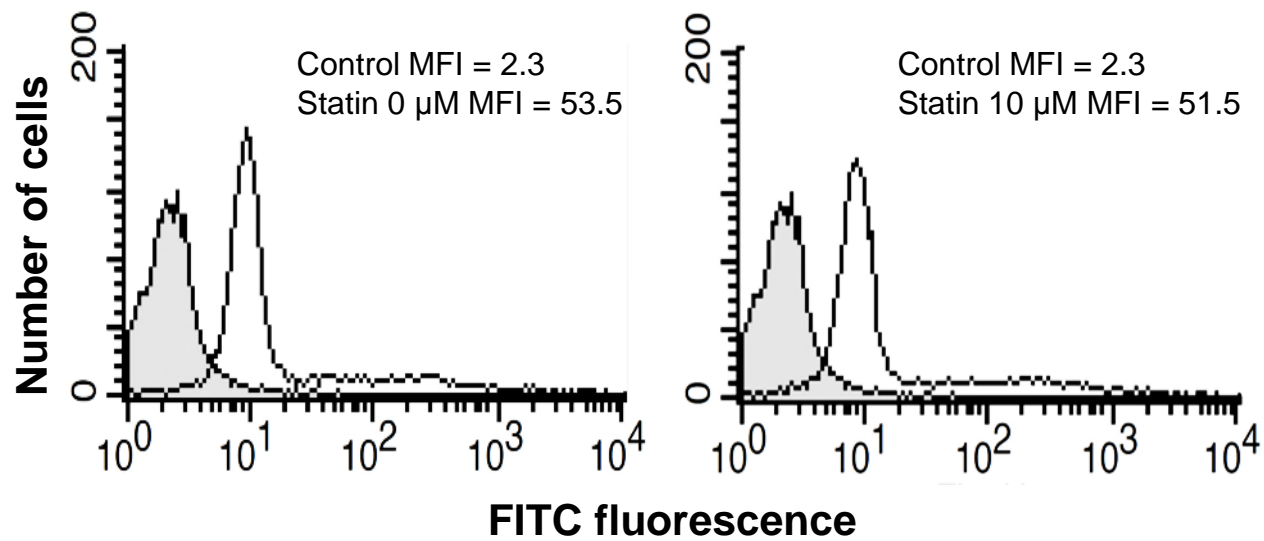

**Fig. S2**

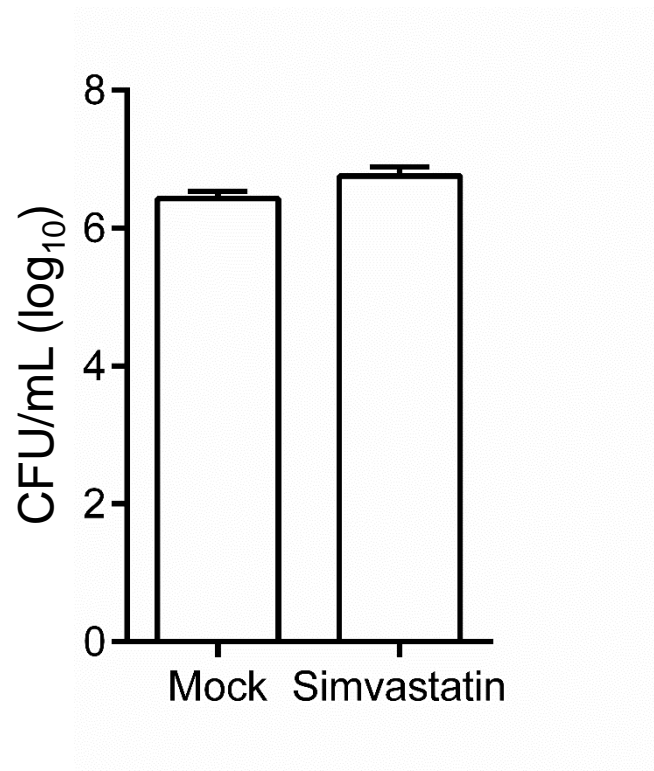

Supplement: Figure S1 — Statin does not affect the phagocytotic activity of macrophages. RAW264.7 cells treated with simvastatin (0 or 10 μM) for 8 h were incubated with latex beads, and phagocytosis activity was evaluated via flow cytometry analysis. The number at the right of each histogram represents the mean fluorescence intensity (MFI). [file Presentation1.PDF]
